# Prevalence and associated factors of abdominal obesity among the adult population in Woldia town, Northeast Ethiopia, 2020: Community-based cross-sectional study

**DOI:** 10.1371/journal.pone.0247960

**Published:** 2021-03-08

**Authors:** Samuel Dagne, Yonatan Menber, Pammela Petrucka, Yosef Wassihun

**Affiliations:** 1 Department of Nutrition and Dietetics, School of Public Health, College of Medicine and Health Sciences, Bahir Dar University, Bahir Dar, Ethiopia; 2 College of Nursing, University of Saskatchewan, Saskatchewan, Canada; 3 Department of Health Promotion and Behavioral Sciences, School of Public Health, College of Medicine and Health Sciences, Bahir Dar University, Bahir Dar, Ethiopia; Yenepoya Medical College, Yenepoya University, INDIA

## Abstract

**Background:**

The prevalence of abdominal obesity has been dramatically increasing both in developed and developing countries, including Ethiopia. It is an independent risk factor for cardiovascular diseases, type-2 diabetes mellitus, high blood pressure, and cancer. However, there is inadequate data regarding the prevalence and associated factors of abdominal obesity among adult population in Ethiopia.

**Objective:**

This study aimed to assess the prevalence and associated factors of abdominal obesity among the adult population in Woldia town, Northeast Ethiopia in 2020.

**Method:**

A community-based cross-sectional study was conducted in Woldia town from March 20 to April 20, 2020. Data on socio-demographic, dietary history, and anthropometric characteristics were collected from 802 adults using the World Health Organization stepwise technique. Multi-stage sampling was employed to select the study subjects. Data were cleaned, coded, and entered by EPI-info Version 7 and were exported to SPSS Version 20 for further analysis. To indicate the strength of association, odds ratios (OR) and 95% confidence intervals (95% CI) were used.

**Result:**

From a total of 823 respondents 802 were involved with a response rate of 97.4%. The overall prevalence of central obesity based on waist to hip ratio was 16.5% with 95% CI (14.2–19.2). Female sex [AOR = 13.3, 95% CI: 7.01–25.39), high wealth rank (AOR = 2.95, 95% CI: 1.21–7.17), single marital status (AOR = 0.16, 95%CI: 0.04–0.58), age from 35 to 55 years (AOR = 4.3, 95% CI: 2.22–7.99), age greater than 55 years (AOR = 3.8, 95%CI: 1.36–10.78), secondary educational level (AOR = 1.83, 95% CI: 1.05–3.18), eat more protein (AOR = 4.22, 95% CI: 1.26–14.22), and consumption of snacks (AOR = 2.78, 95% CI: 1.68–4.61) were significantly associated with abdominal obesity.

**Conclusion:**

The prevalence of abdominal obesity among adults in Woldia town is high, and has become an emerging nutrition-related problem. Being female, older age, being in a high wealth rank, consuming more meat, having secondary education level, and consuming snacks were the risk factors of abdominal obesity. Nutrition interventions should target adults mainly focusing on the alarmingly increase in nutrition problems, such as abdominal obesity, in Ethiopia with specific attention to females.

## Introduction

Obesity is a medical condition characterized by an abnormal fat accumulation which may harm health [1]. Abdominal obesity is one of the main components of metabolic syndrome [2]. It is an independent risk factor for different non-communicable diseases such as; cardiovascular diseases, type-2 diabetes mellitus, high blood pressure, and cancer [3–6]. Obesity and its complications have a significant adverse effect on economic development [7–11].

Each year, 17 million people die prematurely from non-communicable diseases related to preventable factors such as overweight or obesity, tobacco use, unhealthy diet, physical inactivity, and harmful use of alcohol. Of these, 82% are in low- and middle-income countries [12]. By 2030, it is estimated that 38% and 20% of the world adult population will be overweight and obese, respectively [13].

Obesity is a complex health issue resulting from a combination of different factors. Excessive intake of energy-dense foods, physical inactivity, and genetic susceptibility are known causative factors of obesity [14–16]. Currently, there is a nutrition transition in developing countries due to increasing economic development and security. The number of people who are overweight/obese is increasing in part due to adopting a modern lifestyle with less physical activity and excessive consumption of energy-dense foods [17–20].

A systematic review reported that the overall prevalence of central obesity was 41.5% globally. Regarding regional variations, the highest prevalence was found in South America (55.1%), Central America (52.9%), and Africa (49.6%). The prevalence was higher in high-income (41.2%) than low-income countries (27.8%) [21].

A number of studies report prevalence of abdominal obesity as 62.5% in Brazil [15], 37.6% in China [22], 58% in South Africa [23], 24.8% in Tanzania [24], and 67.8% in Sudan [25]. However, there are few studies in Ethiopia on abdominal obesity. A study from Gondar, Northwest Ethiopia reports about 58.5% of adults were centrally obese [26]. Another study from Dilla, South Ethiopia reports prevalence of abdominal obesity as 24.4% [27].

Some literature in Ethiopia indicates overweight/obesity has emerged as a public health problem among adults in Ethiopia particularly in the urban areas [28–31]. However, there is limited literature on abdominal obesity in the Ethiopian context, despite its capacity as an indicator of the risk of different non-communicable diseases (NCDs) with waist circumference (WC) being morepredictive ofmetabolic syndromes, type 2 diabetes, and cardiovascular diseases than body mass index (BMI) [26,32–34].

The primary cause of abdominal obesity is an imbalance between intake and expenditure. Therefore, maintaining a healthy weight and lifestyle modification focusing on improving dietary quality and physical activity is the preferred first-line treatment for its management [5,35,36].

The World Health Organization (WHO) recommends a range of strategies aimed at the reduction of overweight and obesity through healthy eating and physical activity. One of the sustainable development goals (SDGs) targeted by 2030 is to reduce by one-third premature mortality from NCDs through prevention and treatment. But, progress to date has been too slow to meet the global target (10.4% in men and 15.4% in women) [37–39]. Ethiopia has also adopted the national food ad nutrition policy (FNP) aiming to attain optimal nutritional status at all stages of life at a level that is consistent with a high quality of life, productivity, and longevity [40].

Different nutrition programs in Ethiopia give more attention to under nutrition irrespective of the rise of obesity in the country. There is scant information regarding the prevalence and contributing factors of abdominal obesity among adults in Ethiopia as well as in the study area. Thus, this study determined the prevalence of abdominal obesity and associated factors among adult population in Woldia Town, in the northeast region of Ethiopia. The information can be used as baseline evidence for program planners, policymakers, researchers, and organizations who are working on the prevention of chronicNCDs.

## Methods and materials

### Study setting and period

The study was conducted in Woldia town from March 20 to April 20, 2020. The town is located in the Amhara Region, it sits at a latitude and longitude of 11°46′50’N 39°36′0’E, with an elevation 2, 112 meters above sea level. It is 520 km to the north of the capital Addis Ababa. Based on the 2014 national population projection conducted by the Central Statistical Agency of Ethiopia (CSA), Woldia Town has a total population of 180,000, of whom 81,750 are men and 98,250 women [41]. The town administration contains ten kebeles (the smallest administration unit in Ethiopia). The health services include one public hospital, two public health centers, and more than 10 private clinics [42].

### Study design

A community-based cross-sectional study design was employed.

### Inclusion and exclusion criteria

All adults aged 18–64 years who were living in Woldia town for more than six months were eligible for the study. Pregnant women and women who gave birth in the last 6 months, adults with spinal problems, and those who have body deformity around the abdomen, critically ill, and/or unable to communicate were excluded from the study.

### Sample size and sampling procedure

The sample size of the study was computed using Epi-Info Version 7 by considering the following assumptions: prevalence of overweight 58.5% [26], level of significance 95%, 5% margin, and 2 design effect. By adding a 10% non-response rate, the final sample size was 823.

### Sampling procedure

Multistage sampling was used to select the study participants. Initially, four kebeles were selected, of the total ten kebeles, by using the lottery method. The total number of households in each kebele was obtained from the kebele administrators. The total sample size was allocated to each kebele proportionally. Households were selected by systematic random sampling after calculating the sampling interval (K). The starting households in each kebeles were selected by lottery method. When more than one eligible adult was found in the selected households, the lottery method was used to select one of the eligible adults for participation.

### Data collection tools and procedure

The data were collected using interview administered structured and pre-tested questionnaire which included socioeconomic characteristics, dietary history, physical activity, and behavioral characteristics adopted from previous studies [15,26,27,29,43,44] by eight well trained and experienced diploma nurse data collectors and two bachelor prepared nurse supervisors. The global physical activity questionnaire analysis guide [45] and the WHO steps instruments for chronic disease risk surveillance questionnaires were used after some modifications [46].

### Measurements

#### Waist and hip circumference

Waist girth was measured by using a plastic tape to the nearest 0.5 cm placed horizontally midway between the 12th rib and iliac crest on the mid-axillary line. Hip circumference was measured around the widest portion of the buttocks, with the tape parallel to the floor [47]. Waist to hip ratio (WHR) was calculated by dividing WC to hip circumfrence. WHR of >1cm in men and >0.87cm in women were considered as abdominal obesity [47].

#### Assessment of dietary habits

The dietary habits of respondents was assessed using a dichotomous yes and no questionnaire; if the respondent answered yes, then further questions were asked about how frequent per week and month specific food consumption occurred. This questioning included probes regarding their intake of snacks between meals.

Dietary Diversity Score (DDS) was adapted from Food and Nutrition Technical Assistance (FANTA, 2006) indicator guide for measuring household and individual DDS. The DDS was calculated from a single 24-hour recall before data collection. All foods consumed the day before the study were grouped into eight categories and consuming a food from any of the groups was assigned a score of 1 and if no food was taken a score of 0 was given. Adults who had DDS scores ≤3, 4–5, and ≥ 6 were categorized as low, medium, and high DDS, respectively [48].

#### Physical activity

The WHO standard total physical activity calculation guide was used to assess the physical activity level of participants. Sctivity levels were determined according to the three settings (or domains), which included activity at work, travel to and from places and recreational activities, and sedentary behavior. Finally, physical activity was categorized as vigorous physical activity, moderate physical activity, and no exercise (no physical activity) [45].

### Operational definitions

**Dietary Diversity Score (DDS)**
  Low: ≤3 food groups
  Medium: 4–5 food groups
  High: ≥ 6 food groups
**Abdominal obesity**
  Abdominal obese: WHR of >1cm in men and >0.87cm in women
  Normal: WHR ≤ 1cm in men and ≤ 0.87cm in women
**Physical activity**
  Low: No exercise or no physical activity [45]
  Moderate: Low-impact aerobic exercise classes, brisk walking or hiking, and recreational team sports (volleyball, soccer, and so on) [45].
  Vigorous: Running or jogging, high-intensity aerobic classes, competitive full-field sports (soccer), and basketball were considered as physical activity [45].

### Data quality control

The questionnaire was translated into the local language (Amharic) and back to English for consistency. Pre-testing was done within 5% of individuals at a place where the actual data collection was not conducted. Content validation was checked for modified questionnaries by the experts. Data collectors and supervisors were trained for 2 days. On spot-checking and correction were made for incomplete questionnaires by the supervisor. The overall data collection process was overseen by the principal investigator.

### Data analysis

The data was coded, cleaned, and entered into Epi-Info Version 7 and exported to SPSS Version 20 for analysis. Descriptive statistics were computed and the results were reported using tables, figures, and charts. Bi-variable logistic regression was executed and variables with p < 0.25 were fitted to the final multivariable logistic regression to adjust for potential confounders to identify the determinants of central obesity among adults. In the final model, variables with P-value < 0.05 were considered as statistically significant and AOR of 95% CI was used to determine the strength of association. Multicollinearity between the independent variables was also assessed using multiple linear regressions. No evidence of multicollinearity was found as the variance inflation factor (VIF) for all variables was less than ten.

### Ethical considerations and consent to participate

Ethical clearance was obtained from the institutional review board of Woldia University. Permission was obtained from the Woldia town administration Office. Informed verbal consent was obtained from each study subject after the data collectors clearly explained the aims of the study. Respondents were also informed that they can refuse or discontinue participation at any time. Information was recorded anonymously to maintain the confidentiality and privacy of respondents.

## Result

### Socio-demographic and economic characteristics of respondents

A total of 802 adults, with a response rate of 97.4%, were involved in the study. Over half, 418 (52.1%) of the study participants were female and similarly 459 (57.2%) had a family size of more than four members. Nearly two-thirds of 497(61.9%) of respondents were married. Nearly half, 386 (48.2%), of the respondents came from low economicalhouseholds (Table 1).

**Table 1:** Socio-demographic and economic characteristics of adult population in Woldia town, Northeast Ethiopia, 2020.

Variables	Abdominal obese, n(%)	Normal, n(%)	Total frequency, n(%)
**Sex**			
Male	15(3.9)	369(96.1)	384(47.9)
Female	117(28.0)	301(72.0)	418(52.1)
**Age**			
18–24	24(10.5)	205(89.5)	229(28.6)
25–34	32(10.7)	266(89.3)	298(37.2)
35–55	65(27.0)	176(73.0)	241(30.0)
Above 55	11(32.4)	23(67.6)	34(4.2)
**Family size**			
< 4	41(12.0)	302(88.0)	343(42.8)
≥4	91(19.8)	368(80.2)	459(57.2)
**Religion**			
Orthodox	112(17.4)	533(82.6)	645(81.0)
Muslim	17(12.6)	118(87.4)	135(16.8)
Protestant	3(13.6)	19(86.4)	22(2.7)
**Marital status**			
Married	92(18.5)	405(81.5)	497(61.9)
Single	23(9.2)	228(90.8)	251(31.3)
Divorced	10(37.0)	17(63.0)	27(3.4)
Widowed	7(25.9)	20(74.1)	27(3.4)
**Occupation status**			
Merchant	52(17.4)	246(82.6)	298(37.2)
Government worker	40(14.6)	234(85.4)	274(34.2)
NGO	11(18.3)	49(81.7)	60(7.5)
Daily worker	12(19.0)	51(81.0)	63(7.9)
Religious leaders	11(34.3)	21(65.7)	32(3.9)
Drivers	3(25.0)	9(75.0)	12(1.5)
Other**	3(4.8)	60(95.2)	63(7.8)
**Education status**			
No formal education	25(19.7)	102(80.3)	127(15.8)
Primary	8(13.8)	50(86.2)	58(7.2)
Secondary	49(21.4)	180(78.6)	229(28.6)
College and above	50(12.9)	338(87.1)	388(48.4)
**Wealth index**			
High	14(20.0)	56(80.0)	70(8.7)
Middle	63(18.2)	283(81.8)	346(43.1)
Low	55(14.2)	331(85.8)	386(48.2)

** Students and jobless.

### Dietary habits

Nearly all, 782 (97.5%), of the study participants ate cereal-based foods. Similarly, more than half (i.e., 424 (52.9%) and 453 (56.5%)) of the study participants consumed fruits and vegetables one to four times per week, respectively. More than half (53.6%) of study participants did not consume snacks. The majority (697, 86.9%) of study subjects had medium to high DDS (Table 2).

**Table 2:** Dietary habits among adult population in Woldia town, Northeast Ethiopia, 2020.

Variables	Abdominal obesity, n(%)	Normal, n(%)	Total frequency, n(%)
**Cereals**			
Daily	128(16.4)	654(83.6)	782(97.5)
Weekly	2(15.4)	11(84.6)	13(1.9)
Monthly	2(28.6)	5(71.4)	7(0.9)
**Fruits**			
Daily	30(14.7)	174(85.3)	204(25.4)
Weekly	68(16.0)	356(84.0)	424(52.9)
Monthly	23(18.1)	104(81.9)	127(15.8)
Never	11(23.4)	36(76.6)	47(5.9)
**Vegetables**			
Daily	35(15.3)	194(84.7)	229(28.6)
Weekly	76(16.8)	377(83.2)	453(56.5)
Monthly	18(19.8)	73(80.2)	91(11.3)
Never	3(10.3)	26(89.7)	29(3.6)
**Milk and Milk products**			
Daily	47(19.0)	200(81.0)	247(30.8)
Weekly	27(11.4)	209(88.6)	236(29.4)
Monthly	31(16.8)	154(83.2)	185(23.1)
Never	27(20.1)	107(79.9)	134(16.7)
**Fats**			
Daily	20(18.2)	90(81.8)	110(13.7)
Weekly	53(15.4)	291(84.6)	344(42.9)
Monthly	43(15.6)	233(84.4)	276(34.4)
Never	16(22.2)	56(77.8)	72(9.0)
**Meat, egg, and Fish**			
Daily	10(37.0)	17(63.0)	27(3.4)
Weekly	24(16.1)	125(83.9)	149(18.6)
Monthly	27(12.0)	198(88.0)	225(28.0)
Never	71(17.7)	330(82.3)	401(50.0)
**Soft drinks intake**			
Three and more	55(24.1)	173(75.9)	228(28.4)
Twice	23(13.5)	147(86.5)	170(21.2)
Once	27(14.5)	159(85.5)	186(23.2)
Never	27(12.4)	191(87.6)	218(27.2)
**Snack use**			
No	52(12.0)	380(88.0)	432(53.9)
Yes	80(21.6)	290(78.4)	370(46.1)
**Frequency of snack use**			
One times	45(10.9)	368(89.1)	413(95.6)
Two times	7(36.8)	12(63.2)	19(4.4)
**Frequency of meal per day**			
Once	0(0)	3(100)	3(0.4)
Twice	17(10.8)	140(89.2)	157(19.6)
Three times	112(18.2)	503(81.8)	615(76.7)
Four and above	3(11.1)	24(88.9)	27(3.4)
**Dietary Diversity Score (DDS)**			
Low	25(23.8)	80(76.2)	105(13.1)
Medium to high	107(15.4)	590(84.6)	697(86.9)

### Physical activity

The majority of respondents, 675 (84.2%), were engaged in low to moderate workplace activities. However, three-fourths (74.9%) of the study participants had no leisure-time physical activity, and 448 (55.9%) spent three or more hours sitting without any exercise. Nearly half (47%) of the study participants traveled by car (Table 3).

**Table 3:** Physical activities among adult population in Woldia town, Northeast Ethiopia, 2020.

Physical activities	Abdominal obese, n(%)	Normal, n(%)	Total frequency, n(%)
**Workplace physical activity**			
Low	120(17.8)	555(82.2)	675(84.2)
Moderate	11(9.3)	107(90.7)	118(14.7)
Intense	1(11.1)	8(88.9)	9(1.1)
**Walk or use a bicycle at least 30 minutes**			
No	81(18.2)	365(81.8)	446(55.6)
Yes	51(14.3)	305(85.7)	356(44.4)
**Leisure-time physical activity**			
No	118(19.6)	483(80.4)	601(74.9)
Moderate	10(6.0)	158(94.0)	168(21.0)
Intense	4(12.1)	29(87.9)	33(4.1)
**Time spent sitting without any activity**			
< 2 hours per day	35(21.0)	132(79.0)	167(20.8)
2–3 hours per day	29(15.5)	158(84.5)	187(23.3)
> 3 hours per day	68(15.2)	380(84.8)	448(55.9)
**Mode of transport**			
Foot	56(18.5)	246(81.5)	302(37.7)
Car	55(14.6)	322(85.4)	377(47.0)
Both	21(17.1)	102(82.9)	123(15.3)

### Substance use behaviors of respondents

**Fig 1:**
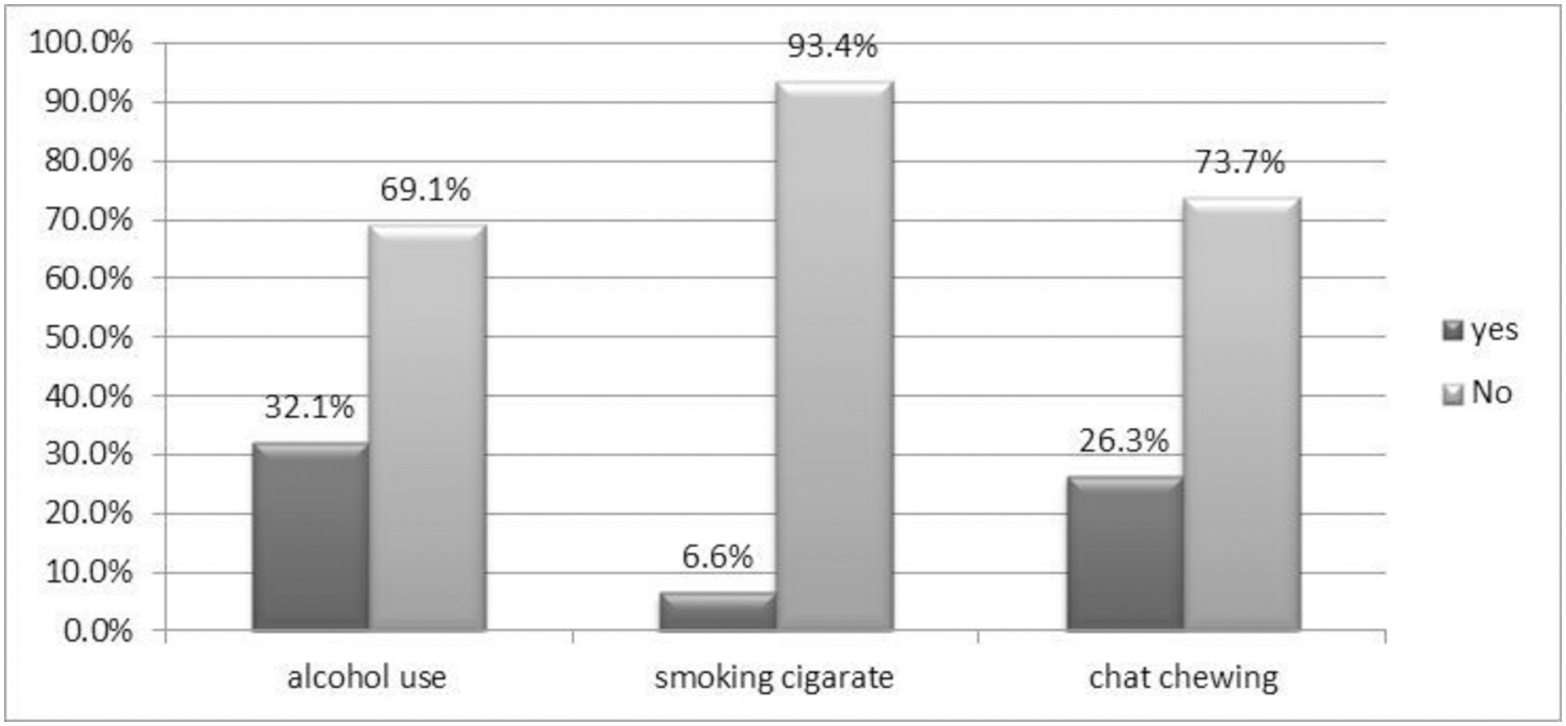
Substance use behavior of adult population in Woldia town, Northeast Ethiopia, 2020.

Regarding substance use behavior, one third (32.1%) of respondents had a habit of drinking alcohol. One fourth (26.3%) of the study subjects chewed chat (Fig 1).

### Prevalence of abdominal obesity

**Fig 2:**
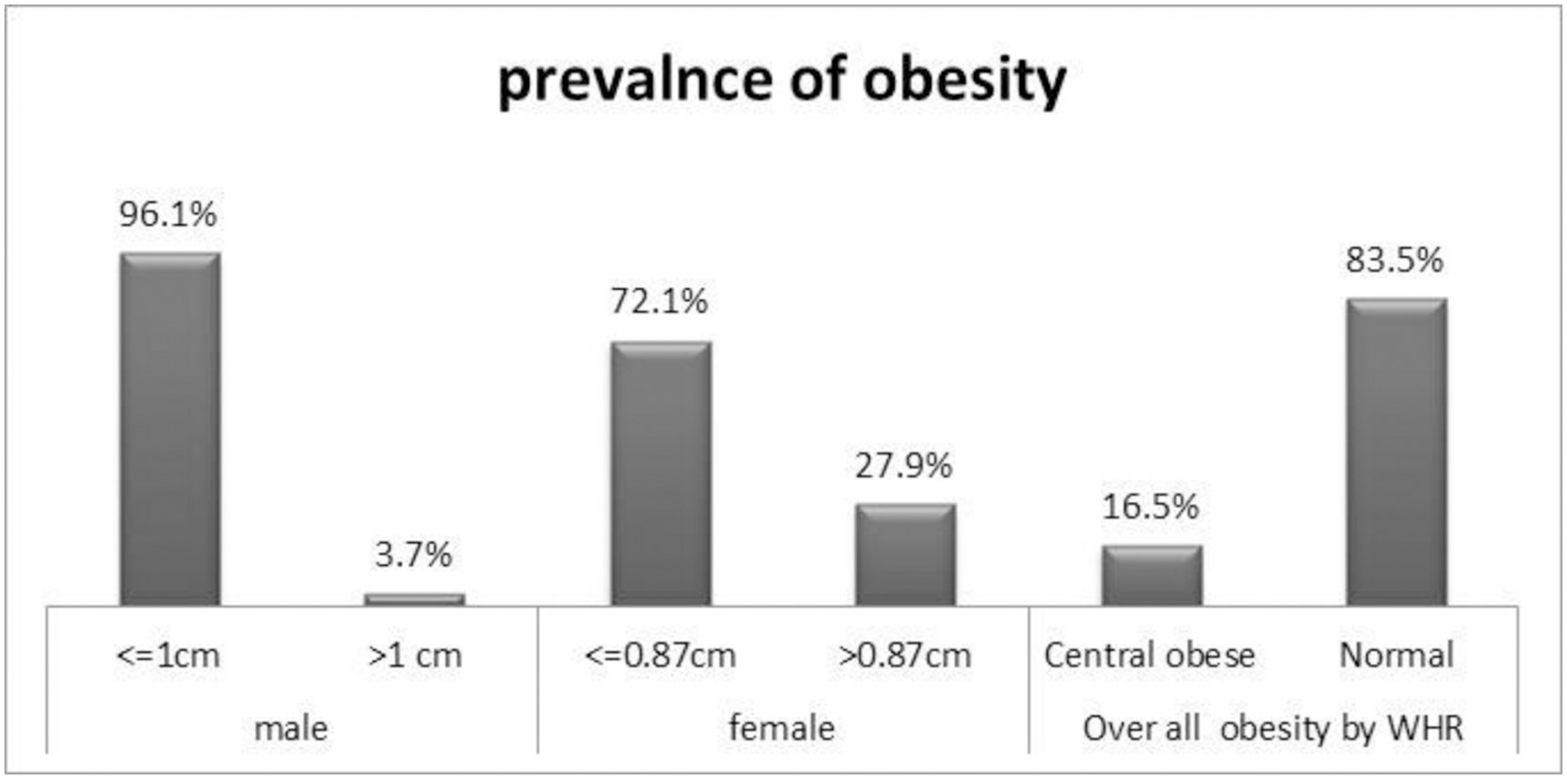
Prevalence of abdominal obesity among adults population in Woldia town, Northeast Ethiopia, 2020.

**Fig 3:**
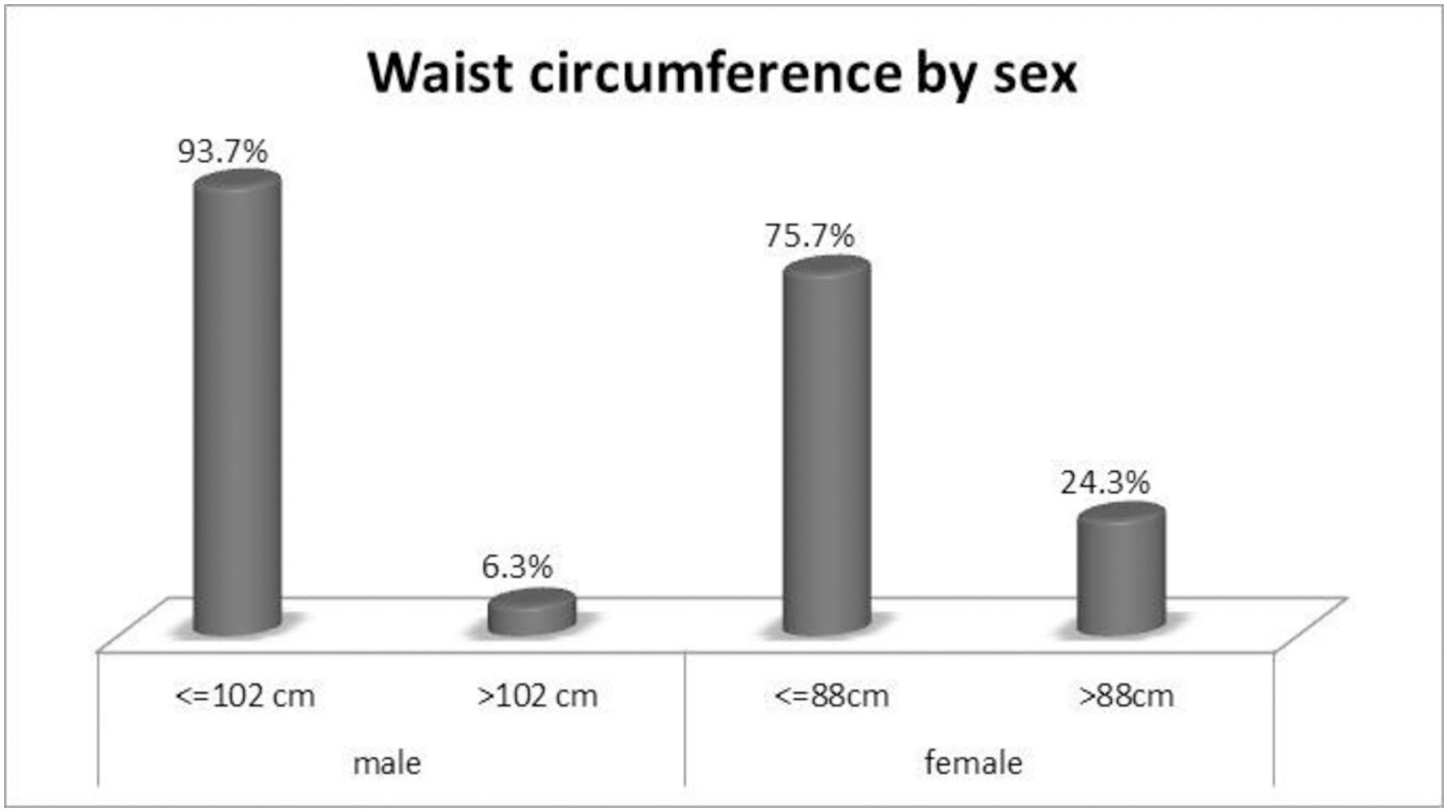
Waist Circumferences by sex among adults population in Woldia town, Northeast Ethiopia, 2020.

The overall prevalence of abdominal obesity based on WHR was 16.5% with 95% CI (14.2–19.2). The prevalence was higher among women (27.9%) than men (3.9%) (Fig 2). RegardingWC, 6.3% of men and 24.3% of women had > 102cm and >88cm, respectively (Fig 3).

### Factors associated with abdominal obesity

In binary logistic regression analysis, sex, age, wealth index, marital status, DDS, educational status, high meat consumption, milk, and milk products intake, soft drink intake, and snack use were significantly associated with abdominal obesity. After controlling for all confounding variables: being female, old age, higher wealth status, single in marital status, secondary school educational level, high meat consumption, and snack use were independent predictors of the outcome variable.

Females were 13.3 times as likely to show abdominal obesity as males (AOR = 13.3, 95% CI: 7.01–25.39). Adults with high wealth rank were 2.9 times more likely to get abdominal obesity than those with low wealth rank (AOR = 2.95, 95% CI: 1.21–7.17). Marital status was another determinant factor for abdominal obesity. The likelihoodof abdominal obesity was 84% less among single than widowed participants (AOR = 0.16, 95%CI; 0.04–0.58). Adults within the 35 to 55 years age group and greater than 55 years were 4.3 (AOR = 4.3, 95% CI; 2.22–7.99) and 3.8 (AOR = 3.8, 95%CI; 1.36–10.78) times more likely to have abdominal obesity than those in the age group of 18–24 years old, respectively. Besides, adults who had secondary educational status were 1.83 times more likely to display abdominal obesity than adults who have college and above level of education (AOR = 1.83, 95%CI: 1.05–3.18). Adults consuming protein-rich foods daily and weekly were 4.2 (AOR = 4.22, 95%CI; 1.26–14.22) and 2.3 (AOR = 2.3, 95%CI; 1.12–4.76) times more likely to be abdominally obese, respectively, than those who never consume protein-rich foods. Furthermore, adults who consumed snacks were 2.8 times more likely to be abdominally obese as compared to adults who never consumedsnacks (AOR = 2.78, 95% CI: 1.68–4.61) (Table 4).

**Table 4:** Risk factors of abdominal obesity among adult population in Woldia town, Northeast Ethiopia, 2020.

Variables	Unadjusted OR (95% CI)	P- value	Adjusted OR (95% CI)	p-value
**Sex**				
Male	1		1	
Female	9.6(5.46–16.72)	<0.001	13.3(7.01–25.39)	<0.001
**Age**				
18–24	1		1	
25–34	1.1(.59–1.79)	0.924	1.16(0.61–2.22)	0.645
35–55	3.2(1.89–5.25)	<0.001	4.3(2.22–7.99)	<0.001
Above 55	4.1(1.78–9.43)	0.001	3.8(1.36–10.78)	0.011
**Wealth Index**				
High	1.51(0.78–2.89)	0.219	2.95(1.21–7.17)	0.017
Middle	1.34(0.91–1.99)	0.147	1.54(0.91–2.59)	0.110
Low	1		1	
**Marital status**				
Married	0.65(0.27–1.58)	0.341	0.41(0.12–1.40)	0.152
Single	0.29(0.11–0.75)	0.011	0.16(0.04–0.58)	0.005
Divorced	1.68(0.53–5.37)	0.381	0.56(0.20–2.64)	0.466
Widowed	1		1	
**Education status**				
No formal education	1.66(0.98–2.81)	0.061	1.69(0.84–3.46)	0.144
Primary	1.08(0.48–2.41)	0.848	0.79(0.34–2.82)	0.969
Secondary	1.84(1.19–2.84)	0.006	1.83(1.05–3.18)	0.032
College and above	1		1	
**DDS**				
Low	1.72(1.05–2.82)	0.031	1.74(0.93–3.15)	0.083
Medium to high	1		1	
**Fat consumption**				
Daily	0.78(0.37–1.63)	0.504	0.91(0.299–2.96)	0.917
Weekly	0.64(0.34–1.19)	0.160	1.29(0.51–3.31)	0.596
Monthly	0.65(0.34–1.23)	0.183	0.86(0.36–2.05)	0.729
Never	1		1	
**Meat, egg, and fish consumption**				
Daily	2.74(1.21–6.22)	0.016	4.22(1.26–14.22)	0.020
Weekly	0.89(0.54–1.48)	0.660	2.3(1.12–4.76)	0.023
Monthly	0.63(0.39–1.02)	0.061	1.25(0.67–2.35)	0.481
Never	1		1	
**Milk and milk products**				
Daily	0.93(0.55–1.58)	0.792	1.13(0.61–2.56)	0.483
Weekly	0.51(0.29–0.92)	0.024	0.69(0.31–1.58)	0.386
Monthly	0.79(0.45–1.41)	0.439	1.87(0.83–4.19)	0.126
Never	1		1	
**Fruit intake**				
Daily	0.56(0.26–1.23)	0.150	0.56(0.19–1.64)	0.287
Weekly	0.63(0.31–1.63)	0.203	0.86(0.32–2.32)	0.770
Monthly	0.72(0.31–1.63)	0.435	0.71(0.25–1.99)	0.510
Never	1		1	
**Soft drinks intake**				
Three and above	2.25(1.36–3.72)	0.002	1.55(0.83–2.86)	0.167
Twice	1.11(0.61–2.01)	0.739	1.04(0.48–2.22)	0.917
Once	1.21(0.68–2.13)	0.531	1.47(0.727–2.96)	0.285
Never	1		1	
**Snack use**				
No	1		1	
Yes	2.02(1.38–2.95)	<0.001	2.78(1.68–4.61)	<0.001
**Walk or use a bicycle at least 30 minutes**				
No	1.33(0.91–1.94)	0.146	1.63(0.99–2.68)	0.053
Yes	1		1	

## Discussion

Abdominal obesity is an independent risk factor for a range of NCDs such as cardiovascular diseases, type-2 diabetes mellitus, high blood pressure, and cancer. It is important to recognized that the associated health problems with NCDs have a significant negative impact on economic development. It is one of the emerging nutritional problems in low and middle-income countries, including Ethiopia. Therefore, the identification of potentially modifiable risk factors for abdominal obesity will potentially help to reduce threats of the problem in developing countries.

In this study, the prevalence of abdominal obesity was 16.5% (95% CI: 14.2–19.2). Being female, old age, higher wealth status, single in marital status, secondary school educational level, more meat consumption, and snack use were significantly associated with abdominal obesity.

In this study, the overall prevalence of central obesity based on WHR was 16.5%. The prevalence was higher among women (27.9%) than men (3.9%). The findings from this study was lower than the report from Gondar (26.7 to 58.5%) [26,43], Dilla (24.4%) [27], Sudan (67.8%) [25], Tanzania (24.88%) [24], West Africa (50.8%) [44], Nigeria (52.6%) [49], South Africa (58%) [23], India (71.2%) [50], Iran (40.7%) [51], and Greece (49.7%) [52]. But, it was higher than a report from China (7.7%) [22]. In this study, abdominal obesity is defined by WHR, which is not consistently used in this area of research and could be a source of variation in prevalence.

In this study, women were about 13 times more likely to have abdominal obesity than their male counterparts. Some studies have reported that abdominal obesity was more common in women compared to men in Sudan [25], Tanzania [24], West Africa [44], South Africa [23], Iran [51], and China [22]. The possible reason for this variation in prevalence could be that the female have more steroid hormones which expose them to obesity [53]. The other possible explanation could be that, in the Ethiopian cultural context, men mainly engaged in activities that require higher energy than women.

Adults aged 35 and aboved were more at risk for abdominal obesity in this study. Similar findings were reported from Tanzania [24], West Africa [44], Iran [51], rural China [54], and Brazil [55] which indicated that abdominal obesity was more common after the age of 40 years old. This observation could be explained in relation to gaining and reduction in physical activities and a propensity for a more sedentary way of life exposing older adults to obesity.

In this study, abdominal obesity was more common among adults from households with a higher wealth status. This finding could be explained in that in developing countries rich adults have improved and predictable access to food, decreased physical activity, and the consumption of "western" diets. The findings on the association between abdominal obesity and wealth status are contradictory. Studies from rural China [54] and West Africa [44] have found that the poor were more likely to experience abdominal obesity than the rich.

Being single was predictive of lower risk for abdominal obesity in this study. Similar findings were reported from studies done in Sudan [25], Tanzania [24], West Africa [44], Brazil [55], and China [54]. This finding could be attributed to a change in eating habits after marriage.

This study revealed that adults who have low educational level were more likely to be abdomenally obese. Reports from West Africa [44], South Africa [23], and Brazil [55] have yielded similar findings to this study. This result could be explained by the fact that people with low levels of education might be exposed to unhealthy diet selection and they are less concerned about the consequences of abdominal obesity. But, studies from Tanzania [24] and rural China [54] showed that adults who have a higher level of education were at high risk for abdominal obesity.

Those adults who consumed more meat were at higher risk of abdominal obesity. Similar findings was reported from rural China [54] and the USA [56], which may possibly relate to the nutrition composition and products (i.e., fats and carbohydrates) of a protein rich diet. This scenariomakes the energy released from protein an excess, which then is converted and stored as extra fat in the human body. The other possible explanation might be in Ethiopian culture after consuming meat food; high alcohol drinking is common for facilitating digestion.

Our investigation showed that adults who consumed snacks were more likely to exhibit abdominal obesity. Similar findings were reported from USA and Italy [57,58]. The possible explanation relates to the high-caloric and low-nutrient content of snack foods.

## Strengths and limitations

The strength of this study is that the prevalence and risk factors associated with abdominal obesity in adults were assessed using representative data. However, it has limitations that need to be taken into consideration. Firstly, the portion size of the food adults consume was not assessed. Another limitation is the variation in WHR cut-point and abdominal obesity definition based on WHO, IDF, and NCEP-ATP III criteria. There might also be recall bias among respondents answering questions related to dietary intake for the month, time spent for physical acivities.

## Conclusion and recommendation

The prevalence of abdominal obesity among adults in Woldia town is high and is an emerging nutrition-related problem. Being female, old age, in the high wealth rank, consuming more meat, holding secondary education level, and consuming snacks were the risk factors associated with abdominal obesity. Nutrition intervention should targets adults mainly focusing on the alarmingly trends in over nutrition dsin Ethiopia with due special attention to females.

## Supporting information

S1 File (DOCX)

S2 File (XLSX)

S3 File (SAV)

## Abbreviations

AOR: Adjusted Odd Ratio
BMI: Body Mass Index
CI: Confidence Interval
CSA: Central Statistics Agency
DDS: Dietary Diversity Score
FNP: Food Nutrition Program
IDA: International Diabetes Federation
NCDs: Non- Communicable Diseases
NCEP- ATP III: National Cholesterol Education Program—Adult Treatment Panel III
SPSS: Statistical Packages for Social Studies
WC: Waist Circumference
WHO: World Health Organization
WHR: Waist to Hip Ratio
