# Supplementary material for: Prevalence and associated factors of abdominal obesity among the adult population in Woldia town, Northeast Ethiopia, 2020: Community-based cross-sectional study
Source: PLoS One. 2021 Mar 8;16(3):e0247960. doi: 10.1371/journal.pone.0247960 (PMC7939297; doi:10.1371/journal.pone.0247960)
Supplement: S1 File — (DOCX) [file pone.0247960.s001.docx]

**Consent Statement**

Questionnaire: For Assessment of abdominal obesity and its associated factors to be filled by adult of Woldia town.

How are you, I am here on behalf of Samuel Dagne, a instructor in Woldia University, college of Health Sciences, department of Public Health.

I would like to ask you a few questions about your socio-demographic status, eating habit, physical exercise, sedentary life style, alcohol use and smoking. And I will measure your waist and hip circumference.

This will help us to improve the prevention and control of abdominal obesity among adults in Woldia town based on the information you provide us. Your name will not be written in this form and will never be used in connection with any information you tell us. All information given by you will be kept strictly confidential. Your participation is voluntary and you are not obligate to answer any question which you do not wish to answer. If you fill discomfort with this, please fill free to drop it any time you want .this will take about 15 minutes. Could I have your permission to continue?

1. If yes, continue

2. If no, skip to the next participant.

Data collectors Name-----------------signature------------------------date--------------------

Thank you!

**English Questionnaire**

Date**………………….**

Participant identification number ……………………………………..

| S. No | Question | Response | Code |
| --- | --- | --- | --- |
| 1 | Name of the kebele | …………………….. | A1 |
| **Socio-demographic profile** | | | |
| 2 | How old are you? | …………….years | B1 |
| 3 | What is your sex? | 1.femal  2.male | B2 |
| 5 | Family size? | ------------------------- | B3 |
| 6 | Religion? | 1.Orthodox  2.Muslim  3.Protestant  4.Catholic  5.other(s)------------------------ | B4 |
| 7 | What is the sex of the head of the household? | 1.Male  2.female | B5 |
| 8 | What is the occupation of the head of the  house hold? | 1.Merchant  2.Employer in government  3.Employer in NGO  4.Daily laborer  5.Faremer  6.other(s) ------------------ | B6 |
| 9 | What is educational status of the head of  household? | 1.Unable to read and write  2. able to read and write  3. primary school  4.secondary school  5.Tertiary education (college and above) | B7 |

| **Wealth index** | | | | | |
| --- | --- | --- | --- | --- | --- |
| **10** | | From materials listed below which one is found in your house. | yes | No | C1 |
| A | | Car | yes | No |  |
| A | | Bicycle | yes | No |  |
| C | | Motor cycle | yes | No |  |
| D | | Table | yes | No |  |
| E | | Chair | yes | No |  |
| F | | Sofa | yes | No |  |
| G | | Television | yes | No |  |
| H | | Mobile phone | yes | No |  |
| I | | House phone | yes | No |  |
| J | | Refrigerator | yes | No |  |
| K | | Electric stove | yes | No |  |
| L | | Radio | yes | No |  |
| M | | Horse wheel | yes | No |  |
| 11 | | Floor type | 1. Cement 2. Wood 3. Mud 4. Other------- | | C2 |
| 12 | | Wall type | 1. cement  2. blocket  3. mud  4. other | | C3 |
| 13 | | Type of roof | 1. Can 2. concrete 3. other | | C4 |
| 14 | Owner ship of your house | | 1. rent 2. privet 3. free 4. other | | C5 |

| 15 | | House hold food security | |  | | | | Yes | | No | C6 |
| --- | --- | --- | --- | --- | --- | --- | --- | --- | --- | --- | --- |
|  |  |  |  | Go whole day without food | | | | Yes | | No |  |
|  |  |  |  | Worry about food | | | | Yes | | No |  |
|  |  |  |  | Inability to eat preferred food | | | | Yes | | No |  |
|  |  |  |  | Limited variety of food | | | | Yes | | No |  |
|  |  |  |  | Inability to access to preferred | | | | Yes | | No |  |
|  |  |  |  | Smaller amounts of foods | | | | Yes | | No |  |
|  |  |  |  | Reduced number of meals | | | | Yes | | No |  |
|  |  |  |  | Ever no enough food | | | | Yes | | No |  |
|  |  |  |  | Sleep hungry | | | | Yes | | No |  |
| **Dietary habit** | | | | | | | | | | | |
| 16 | | | How many days per week did you usually  eat fruit such as orange, banana | | 1. Two to four times per day  2. Once per day  3. Two to four times per week  4. Once per week  5. Two to four times per month  6.Once per month  7. Never | | | | | | D1 |
| 17 | | | How many days per week did you usually  eat vegetables? | | 1.Two to four times per day  2.Once per day  3. Two to four times per week  4.Once per week  5.Two to four times per month  6.Once per month  7.never | | | | | | D2 |
| 18 | | | How often did you usually eat milk,  yoghurt and cheese? | | 1.Two to four times per day  2.Once per day  3. Two to four times per week  4.Once per week  5.Two to four times per month  6.Once per month  7.Never | | | | | | D3 |
| 19 | | | How often did you usually eat  meat, egg and fish? | | 1.Two to four times per day  2.Once per day  3. Two to four times per week  4.Once per week  5.Two to four times per month  6.Once per month  7.Never | | | | | | D4 |
| 20 | | | How often did you usually  eat beans? | | 1.Two to four times per day  2.Once per day  3. Two to four times per week  4.Once per week  5.Two to four times per month  6.Once per month  7.Never | | | | | | D5 |
| 21 | | | How often did you usually eat sweets and  sugars? | | 1.Two to four times per day  2.Once per day  3. Two to four times per week  4.Once per week  5.Two to four times per month  6.Once per month  7.Never | | | | | | D6 |
| 22 | | | How often did you usually eat meat, egg  and fish? | | 1.Two to four times per day  2.Once per day  3. Two to four times per week  4.Once per week  5.Two to four times per month  6.Once per month  7.Never | | | | | | D7 |
| 23 | | | Do you have a snack currently? | | 1.Yes  2.No if no go to D11 | | | | | | D8 |
| 24 | | | If your answer is yes what do you have for snack? | | ……………………….. | | | | | | D9 |
| 25 | | | How many times a day do you have snack? | | 1.One times  2.Two times  3.Three times and above | | | | | | D10 |
| 26 | | | How often do you serve meals per day  other than snack | | 1.One times  2.Two times  3.Three times  4.Four and above | | | | | | D11 |
| 27 | | | List foods that usually bought  in addition to regular meal? | | 1-cake  2-biscuit  3-ice cream  4-chocolate  5-others specify……… | | | | | | D12 |
| 28 | | | Do you eat when you watch television/movies? | | 1-yes  2-no  3-i did not watch television/movies | | | | | | D13 |
| 29 | | | How often do you drink soft drinks like  coca, Pepsi per week? | | 1. i do not drink 2. one times 3. two times 4. three times and above | | | | | | D14 |
| 30 | | | How many varieties food you consume in the  last 24 hours? | | 1. less than four  2. four up eight  3. greater than eight | | | | | | D15 |
| 31 | | | Do you fast currently? | | 1.Yes  2.no | | | | | | D16 |
| 32 | | | For what reason do you fast? | | 1. For decrease weight 2. For religious purpose 3. Other | | | | | | D17 |
| **Physical activity** | | | | | | | | | | | |
| 33 | | | Do you engaged in work that involve  moderate to vigorous –intensity activity  Besides your education? | | | | | | 1-yes  2-no  If no go to E3 | | E1 |
| 34 | | | If your answer in E1 is yes, how many  days do your work –in a week | | | | | | Number of days ………….. | | E2 |
| 35 | | | Do you walk or use a bicycle for  at least 30 minutes continuously to  get and from places? | | | | | | 1-yes  2-no  If no go to E5 | | E3 |
| 36 | | | If yes to E3, how many day do you walk  or use a bicycle for at least 30 minutes  Continuously per week? | | | | | | …………………….. | | E4 |
| 37 | | | Do you do any vigorous –intensity  sports that cause large increase in  breasting or heart rate for at least  10 minutes continuously? | | | | | | 1-yes  2-no  If no go to E8 | | E5 |
| 38 | | | In a week on how many days do you do vigorous-intensity sports, fitness  or recreational activities? | | | | | | Number of days …………… | | E6 |
| 39 | | | How much time do you spend doing  vigorous-intensity sports, fitness  or recreational activities in a day? | | | | | | Hours: minutes…………… | | E7 |
| 40 | | | Do you do any moderate –intensity  sports that cause small increase in  breathing or heart rate at least  10 minutes continuously? | | | | | | 1-yes  2-no  If no go to E11 | | E8 |
| 41 | | | In a week on how many days do  you do moderate-intensity  sports, fitness or recreational activity? | | | | | | ………………………… | | E9 |
| 42 | | | How much time do you spend doing  moderate- intensity sports, fitness or recreational activities in a day ? | | | | | | Hours: minutes…………… | | E10 |
| 43 | | | How much time do you get to and from  school? | | | | | | 1-on foot  2-by taxi or service(bus) | | E11 |
| 44 | | | How much time do you usually spend  watching TV/film in a day | | | | | | Hours; | | E12 |
| 45 | | | How much time do you usually spend  sitting when you are reading in a day | | | | | | Hours; | | E13 |
| **Alcohol use and smoking behavior** | | | | | | | | | | | |
| 46 | Do you drink alcohol like  beer, wine currently? | | | | | | 1,yes  2,no if no go to F3 | | | | F1 |
| 47 | If yes how often do you drink  per week? | | | | | | 1-one day  2-two day  3-three day  4-four days and above | | | | F2 |
| 48 | Do you smoke currently? | | | | | | 1-yes  2-no | | | | F3 |
| 49 | If yes, how often do you smoke? | | | | | | 1-daily  2-occasionally | | | | F4 |
| 50 | Do you chew chat? | | | | | | 1-yes  2-no | | | | F5 |
| 51 | If you say yes, how often do you chew  Chat per week? | | | | | | 1. One day 2. Two day 3. Three day 4. four and above | | | | F6 |
|  | **Heath related** | | | | | |  | | | |  |
| 52 | Have you ever been used hormonal family methods? | | | | | | 1. Yes, if yes for how long? --------  2.no | | | | G1 |
| 53 | Are/were your parents (first degree relatives) are / were  Overweight / obese? | | | | | | 1.yes  2.no | | | | G2 |
| 54 | If yes, who are /were obese? | | | | | | 1.mother  2.father  3. brother  4. sister | | | | G3 |
| 55 | Have you ever been got depression/anxiety in your life? | | | | | | 1. Yes, if yes for how long? --------  2. no | | | | G4 |
| 56 | Do you take drugs for a long period of time? | | | | | | 1.yes  2.no | | | | G5 |
| 57 | If yes, what type drug? | | | | | | ----------------------------------------------------- | | | | G6 |
|  | **Knowledge and perception** | | | | | |  | | | |  |
| 58 | Do you know the causes of overweight and obesity? | | | | | | 1.yes  2.no | | | | H1 |
| 59 | If yes, mention causes | | | | | | 1.-------------------------  2.-------------------------  3.…………………… | | | |  |
| 60 | Can you mention some complications  of overweight and obesity? | | | | | | 1. …………………..  2.……………………  3.------------------------- | | | | H2 |
| 61 | Do you think being overweight and obesity good for  You? | | | | | | 1.yes  2. no | | | | H3 |
| **Physical measurements** | | | | | | | | | | | |
| 62 | | | Waist circumference | | | In centimeters--------------- | | | | | I1 |
| 63 | | | Hip circumference | | | In centimeters -------------- | | | | | I2 |

**የቦርጭ ተያያዥ ሁኔታዎች የምርምር ወይም ጥናታዊ ፅሁፍ መሰብሰቢያ የተዘጋጀ መጠይቅ**

**የስምምነት ቃል**

ሰ ላምታ፡ እንደምነህ/ነሽ

ስሜ ይባላል፡፡ እዚህ የተገኘጉት አቶ ሳሙኤል ዳኘ በወልድያ ዩኒቨርሰቲ ጤና ሳይንስ ፋካልቲ የህብረተሰብ ጤና አጠባበቅ ትምህርት ቤት መምህር የሆኑትን ወክዬ ነው፡፡

ለምናጠናው ጥናት የተወሰኑ ጥያቄዎችን እጠይቅሃለሁ/እጠይቃሻለሁ፡፡ በዚህ መጠይቅ የማህበራዊና ስነ-ህዝባዊ ሁኔታ፣ የአመጋገብ ልምድ፣ ስለአካላዊ እንቅስቃሴ፣ በመቀመጥ የምታሳፈውን/የምታሳልፊውን ጊዜ፣ ስለአልኮል መጠጠና ሲጋራ ማጨስ በተመለከተ እጠይቃለሁ/ እጠይቅሻለሁ፡፡ ከአንተ/ች የምናገኘው መረጃ፤ ከቦርጭ ጋር ተያይዘው የሚመጡ የጤና ችግሮች ለመከላከልና እቅድ ለማውጣት ይረዳናል፡፡ ከአንተ/ች የምናገኘውን መረጃ በምሥጢር እንጠብቃለን፡፡ ከአንተ/ች የምንሰበስበው መረጃ ከአንተ/ች ስም ጋር አይያያዝም፡፡

መልስ መስጠት የማትፈልግበት/የማትፈልጊበት ጥያቄ ካለ አትገደድም አትገደጅም፡፡ ደስ ካላለህ/ሽ በማንኛውም ጊዜ ማቆም ትችላለህ/ትችያለሽ፡፡ ቃለ መጠየቁ 15 ደቂቃ ሊወስድ ይችላል፡፡

በዚህ መሰረት በጥናቱ ለመሳተፍ ፈቃደኛ ነህ/ነሽ ---------------------------------

መልሱ ፈቃደኛ ነኝ ከሆነ ወደ ቀጣዮች ጥያቄዎች ይህዱ

የመረጃ ሰብሳቢው ስም--------------------------------------- ፊርማ-------------------- ቀን--------------**አመሰግናለሁ!!!**

**¾›T`— SÖÃp**

¾}dታò¨< **SKÁ lØ\**

| **¡õM ›”É:-** ¾kuK?¬ eU | | | | |
| --- | --- | --- | --- | --- |
| **}.l** | **SÖÃp** | | **SMe** | **¢É** |
| 1 | ¾kuK?¬ eU | | _______________ | A1 |
| **¡õM G<Kƒ:- TIu^©“ e’-I´v© S[Í** | | | | |
| 2 | °ÉT@ | ...............¯Sƒ | | B1 |
| 3 | ïታ | 1. 1. ¨”É 2. c?ƒ | | B2 |
| 4 | ¾u?}cw w³ƒ | --------------------------- | | B5 |

| 5 | HÃT•ƒ | 1. 1. ኦርቶዶክስ ክርስትያን 3. ኘሮቴስታንት 2. 2. ሙስሊም 4. ካቶሊክ 3. 5. ሌላ ከሆነ ይጥቀሱ --------- | B6 |
| --- | --- | --- | --- |

| 6 | ¾u?}cu< S] ïታ | 1. ¨”É2. c?ƒ | B7 |
| --- | --- | --- | --- |
| 7 | ¾u?}cu< S] e^ | 1. ገበሬ 4. ነጋዴ  2. የቀን ሰራተኛ 5. K?L ÃØkc< -------------  3. የመንግሰትc^}— | B8 |
| 8 | ¾u?}cc< S] ¾ƒUI[ƒ Å[Í | 1. T”uw“ Síõ ¾T>‹M 4 . G<K}— Å[Í   1. 2. T”uw“ Síõ ¾TÃ‹M 5 .¢K?Ï“ Ÿ²=Á uLÃ 2. 3. ›”Å— Å[Í | B9 |

| 9 | ¾u?}cw U×’@ Gwƒ | | | | | | | | | | | | | | | | | | | | | | C1 | |  |
| --- | --- | --- | --- | --- | --- | --- | --- | --- | --- | --- | --- | --- | --- | --- | --- | --- | --- | --- | --- | --- | --- | --- | --- | --- | --- |
|  | _ÉÄ | | | | | | | | | | | | 1.›­ 2. ¾KU | | | | | | | | | |  |  |  |
|  | ቴK?y=»” | | | | | | | | | | | | 1.›­ 2. ¾KU | | | | | | | | | |  |  |  |
|  | ¾u?ƒ eM¡ | | | | | | | | | | | | 1.›­ 2. ¾KU | | | | | | | | | |  |  |  |
|  | ¾VvÃM eM¡ | | | | | | | | | | | | 1.›­ 2. ¾KU | | | | | | | | | |  |  |  |
|  | Tk´k¹ õ]Ï | | | | | | | | | | | | 1.›­ 2. ¾KU | | | | | | | | | |  |  |  |
|  | Ö[â?³ | | | | | | | | | | | | 1.›­ 2. ¾KU | | | | | | | | | |  | |  |
|  | ¨”u` | | | | | | | | | | | | 1.›­ 2. ¾KU | | | | | | | | | |  |  |  |
|  | ¾›?K?¡ƒ]¡ Sw^ƒ | | | | | | | | | | | | 1.›­ 2. ¾KU | | | | | | | | | |  |  |  |
|  | ¾›?K?¡ƒ]¡ U×É | | | | | | | | | | | | 1.›­ 2. ¾KU | | | | | | | | | |  |  |  |
|  | we¡K?ƒ | | | | | | | | | | | | 1.›­ 2. ¾KU | | | | | | | | | |  |  |  |
|  | V}` dÃ¡M | | | | | | | | | | | | 1.›­ 2. ¾KU | | | | | | | | | |  |  |  |
|  | ¾ð[e Ò] | | | | | | | | | | | | 1.›­ 2. ¾KU | | | | | | | | | |  |  |  |
|  | SŸ=“ | | | | | | | | | | | | 1.›­ 2. ¾KU | | | | | | | | | |  |  |  |
|  | ሶó | | | | | | | | | | | | 1.›­ 2. ¾KU | | | | | | | | | |  |  |  |
| 10 | ¾T>•\uƒ u?ƒ  vKu?ƒ’ƒ ¾T” ’¨<; | | | | | | 1. ¾^e 3. ’í  2. Ÿ=^Ã 4. K?L ÃØkc< --------------------- | | | | | | | | | | | | | | | | C2 | |  |
| 11 | ¾u?ƒ­ ¨KM ¾}c^¨<  ŸU”É” ’¨<; | | | | | | 1. ›ð`&›iª&ßn 3. ”Úƒ/×¨<L&k`nH 2. Ÿc=T>”„ 4. K?LÃØkc<----------------- | | | | | | | | | | | | | | | | C3 | |  |
| 12 | ¾u?ƒ­ÓÉÓÇ ¾}c^¨<  ŸU”É” ’¨<; | | | | | | 1. ßn&”Úƒ& ካ`„”&g”uq  2. Ÿc=T>”„/wKAŸ?ƒ  3. uvIL© S”ÑÉ ŸÉ”ÒÃ ¾}c^  4. K?LÃØkc ----------- | | | | | | | | | | | | | | | | C4 | |  |
| 13 | ¾u?ƒ­ ×]Á ¾}c^¨<  ŸU”É” ’¨<; | | | | | | 1. q`qa 2. c=T>”„ 3. K?LÃØkc< -------------- | | | | | | | | | | | | | | | | C5 | |  |
| 14 | ቤተሰብዎለመፀዳጃነት የሚጠቀመዉ የትኛዉን የሽንት ቤት አይነት ነዉ; | | | | | | 1. ¾}hሻK ¾Ñ<É¹É 3. ÁM}hhK ¾Ñ<É¹É 2. u¬H ¾T>ìÇ 4. K?L (ÃØkc<)------------- | | | | | | | | | | | | | | | | C6 | |  |
|  | **¡õM Zeƒ:- ¾›SÒÑw MUÉ** | | | | | |  | | | |  | | | | | | | | | | | |  | |  |
| 15 | ›w³—¨<” Ñ>²? ŸIKA‹ና(ጤፍ፣ማሽላ..) ŸØ^Ø_­‹(አተር፣ ምስር፣ሽምብራ…) ¾}²ÒÌ UÓx‹” ue”ƒ Ñ>²? ¨<eØ ÃSÑvK<; | | | |  | | | | 1. Ÿ2 - 4 Ñ>²? uk” **5**. Ÿ1 - 3 Ñ>²? u¨` 2. 1 Ñ>²? uk” 6. 1 Ñ>²? u¨` 3. Ÿ2 - 4 Ñ>²? udU”ƒ 7. ›MSÑwU 4. 1 Ñ>²? udU”ƒ | | | | | | | | | | | | | | D1 | |  |
| 16 | ›w³—¨<” Ñ>²? ፍራፍሬወችን ue”ƒ Ñ>²? ¨<eØ ÃSÑvK<; | | | |  | | | | 1. Ÿ2 - 4 Ñ>²? uk” **5**. Ÿ1 - 3 Ñ>²? u¨` 2. 1 Ñ>²? uk” 6. 1 Ñ>²? u¨` 3. Ÿ2 - 4 Ñ>²? udU”ƒ 7. ›MSÑwU 4. 1 Ñ>²? udU”ƒ | | | | | | | | | | | | | | D2 | |  |
| 17 | ›w³—¨<” Ñ>²? ›ƒ¡M„‹”/ ›[”¹È pÖL pÖKA‹” ue”ƒ Ñ>²? ¨<eØ ÃSÑvK<; | | | |  | | | | 1. 1. Ÿ2 - 4 Ñ>²? uk” **5**. Ÿ1 - 3 Ñ>²? u¨` 2. 2. 1 Ñ>²? uk” 6. 1 Ñ>²? u¨` 3. 3. Ÿ2 - 4 Ñ>²? udU”ƒ 7. ›MSÑwU 4. 4. 1 Ñ>²? udU”ƒ | | | | | | | | | | | | | | D3 | |  |
| 18 | ›w³—¨<” Ñ>²? ¨}ƒ“ ¾¨}ƒ }ªî±­‹” ue”ƒ Ñ>²? ¨<eØ ÃSÑvK<; | | | |  | | | | 1. 1. Ÿ2 - 4 Ñ>²? uk” **5**. Ÿ1 - 3 Ñ>²? u¨` 2. 2. 1 Ñ>²? uk” 6. 1 Ñ>²? u¨` 3. 3. Ÿ2 - 4 Ñ>²? udU”ƒ 7. ›MSÑwU 4. 4. 1 Ñ>²? udU”ƒ | | | | | | | | | | | | | | D4 | |  |
| 19 | ›w³—¨<” Ñ>²? eÒና እንቁላልና አሳ ue”ƒ Ñ>²? ¨<eØ ÃSÑvK<; | | | |  | | | | 1. 1. Ÿ2 - 4 Ñ>²? uk” **5**. Ÿ1 - 3 Ñ>²? u¨` 2. 2. 1 Ñ>²? uk” 6. 1 Ñ>²? u¨` 3. 3. Ÿ2 - 4 Ñ>²? udU”ƒ 7. ›MSÑwU 4. 4. 1 Ñ>²? udU”ƒ | | | | | | | | | | | | | | D5 | |  |
| 20 | ›w³—¨<” Ñ>²? አተር እና ባቄላና የመሳሰሉትንue”ƒ Ñ>²? ¨<eØ ÃSÑvK<; | | | |  | | | | 1. 1. Ÿ2 - 4 Ñ>²? uk” **5**. Ÿ1 - 3 Ñ>²? u¨` 2. 2. 1 Ñ>²? uk” 6. 1 Ñ>²? u¨` 3. 3. Ÿ2 - 4 Ñ>²? udU”ƒ 7. ›MSÑwU 4. 4. 1 Ñ>²? udU”ƒ | | | | | | | | | | | | | | D6 | |  |
| 21 | ›w³—¨<” Ñ>²? ስካE`ና ሌሎች ጣፋጭ ምግቦችንue”ƒ Ñ>²? ¨<eØ ÃSÑvK<; | | | |  | | | | 1. 1. Ÿ2 - 4 Ñ>²? uk” **5**. Ÿ1 - 3 Ñ>²? u¨` 2. 2. 1 Ñ>²? uk” 6. 1 Ñ>²? u¨` 3. 3. Ÿ2 - 4 Ñ>²? udU”ƒ 7. ›MSÑwU 4. 4. 1 Ñ>²? udU”ƒ | | | | | | | | | | | | | | D7 | |  |
| 22 | ›w³—¨<” Ñ>²? ስባማና ጮማ ምግቦችን ue”ƒ Ñ>²? ¨<eØ ÃSÑvK<; | | | |  | | | | 1. 1. Ÿ2 - 4 Ñ>²? uk” **5**. Ÿ1 - 3 Ñ>²? u¨` 2. 2. 1 Ñ>²? uk” 6. 1 Ñ>²? u¨` 3. 3. Ÿ2 - 4 Ñ>²? udU”ƒ 7. ›MSÑwU 4. 4. 1 Ñ>²? udU”ƒ | | | | | | | | | | | | | | D8 | |  |
| 23 | S¡ce (TqÁ) ÃÖkTK< ; | | | |  | | | | 1. ›­2. ¾KU&SMc< ›ÃÅKU ŸJ’ ¨Å D12ÃH>Æ | | | | | | | | | | | | | | D9 | |  |
| 24 | ŸLÃ K}Ökc¨< ØÁo SMe­ ›­ ŸJ’ w²< Ñ>²? ŸU” ›Ã’ƒ UÓw ¾}c^ S¡ce ’¨< ¾T>ÖkS<ƒ; | | | |  | | | | | ____________________________ | | | | | | | | | | | | | D10 | |  |
| 25 | uk” e”ƒ Ñ>²? S¡ce ÃÖkTK<; | | | |  | | | | 1. ›”É Ñ>²? 2. G<Kƒ Ñ>²?3. Zeƒ Ñ>²?“ Ÿ³ uLÃ | | | | | | | | | | | | | | D11 | |  |
| 26 | ŸLÃ ¾Ökc¨<” UÓw (S¡ce) dÃÚU` uk” Ke”ƒ Ñ>²? ÃSÑvK<; | | | |  | | | | 1. ›”É Ñ>²? 2. G<Kƒ Ñ>²?  3. Zeƒ Ñ>²? 4. ›^ƒ“ Ÿ³ uLÃ | | | | | | | | | | | | | | D12 | |  |
| 27 | SÅu— ŸT>SÑu<ƒ UÓw u}ÚT] ¾T>ÖkS<›†¬” UÓx‹ ²`´\ | | | |  | | | | 1. 1. Ÿ?¡ 3 .weŸ<ƒ 2. 2. ›Ãe ¡_U 4. †¢K?ƒ 3. 5. K?L ÃÑKî ------ | | | | | | | | | | | | | | D13 | |  |
| 28 | +y=/òMU uT>SKŸ~ Ñ>²? UÓw ÃSÑvK< | | | |  | | | | 1. ›­ 2. ¾KU 3. +y= ›MSKŸƒU | | | | | | | | | | | | | | D14 | |  |
| 29 | KeLd SÖÙ‹”( üýሲ፣ኮካ) ምን ያክል ጊዜ ይጠቀማሉ; | | | |  | | | | 1. አልጠቀምም 3. አንድ ጊዜ  2. ሁለት ጊዜ 4. ሶስት ጊዜ እና ከዚያ በላይ | | | | | | | | | | | | | | D15 | |  |
| 30 | ላለፉት 24 ሰዓት ዉሰጥ ስንት አይነት የምግብ ዓይነቶችን ተጠቅመዋል; | | | |  | | | | 1. 1. ከ አራት ያነሰ 3. ከ አራት እስከ ስምንት 2. 2. ከስምንት በላይ | | | | | | | | | | | | | | D16 | |  |
| 31 | ïU ÃïTK<; | | | |  | | | | 1. 1.›­ 2. ¾KU | | | | | | | | | | | | | | D17 | |  |
| 32 | SMe­ ›­ ŸJ’ KU”  ¯LT ’¨< ¾T>ïS<ƒ; | | | |  | | | | 1. 1. ¨<õ[ƒ” KSk’e 2. KGÃT•© ¯LT   3.K?L ---------------- | | | | | | | | | | | | | | D18 | |  |
| 33 | የቤተሰብ  የምግብ ዋስትና | | ሀ | k’<” S<K< ÁK UÓw Ã¨<LK<; | | | | | | | | | | | | | | | 1.አዎ | 2.የለም | | | D19 | |  |
|  |  |  | ለ | eK UÓw TÓ–ƒ ›K TÓ–ƒ }Ú”k¨< Á¨<nK<; | | | | | | | | | | | | | | | 1.አዎ | 2.የለም | | |  |  |  |
|  |  |  | ሐ | KSSÑw ¾S[Ö<ƒ” UÓw G<K?U }SÓu¨< Á¨<nK<; | | | | | | | | | | | | | | | 1.አዎ | 2.የለም | | |  |  |  |
|  |  |  | መ | ¾}¨c’< ¾UÓw ¯ይ’„‹” w‰ ’¨Ã ¾T>SÑu<ƒ; | | | | | | | | | | | | | | | 1.አዎ | 2.የለም | | |  |  |  |
|  |  |  | ሠ | ¾T>ðMÑ<ƒ” UÓw ukLK< ÁÑ—K <(ŸÑuÁ ¨²}...) | | | | | | | | | | | | | | | 1.አዎ | 2.የለም | | |  |  |  |
|  |  |  | ረ | ¾T>SÑu<ƒ UÓw ŸT>eðMÓ­ƒ ›ንí` c=ታÃ uSÖ”›’e}—’¨<; | | | | | | | | | | | | | | | 1.አዎ | 2.የለም | | |  |  |  |
|  |  |  | ሰ | uk” UÓw ¾T>SÑu<uƒ Ñ>²? w³ƒ ŸT>eðMÓ­ƒ ›ንí` c=ታÃ ulØ` ›’e}— ’¨<; | | | | | | | | | | | | | | | 1.አዎ | 2.የለም | | |  |  |  |
|  |  |  | ሸ | በቤት ውስጥ ምግብ በጭራሽ ጠፍቶ ያውቃል | | | | | | | | | | | | | | | 1.አዎ | 2.የለም | | |  | |  |
|  |  |  | ቀ | እየራቦዎት ተኝተው ያውቃሉ | | | | | | | | | | | | | | | 1.አዎ | 2.የለም | | |  | |  |
| **¡õM ›^ƒ:- ›"L© ”penሴ** | | | | | | | | | | | | | | | | | | | | | | | | |  |
| 34 | | Ÿስራ­ ¨ß S"ŸK— ¨ÃU w`~ Ñ<Muƒ ¾T>ÖÃp e^ Ãc^K<; | | | | | | | | | | | | | 1.›­ 2. ¾KU SMc< ¾KU ŸJ’ ¨Å E3 | | | | | | | | E1 | |  |
| 35 | | ከዚህ በላይ ለተጠቀሰው ጥያቄ(E1) መልስዎ አዎ ከሆነ በሳምንት ስንት ጊዜ ይሰራሉ? | | | | | | | | | | | | | የቀን ብዛት------------------ | | | | | | | | E2 | |  |
| 36 | | ከቦታ ቦታ በሚንቀሳቀሱበት ጊዜ ለ30 ደቂቃ ያለማቋረጥ በእግርዎ ወይም በብስክሌት ይሄዳሉ? | | | | | | | | | | | | | 1. አዎ 2. አይደለም መልሱ አይደለምከሆነወደE5 ይሂዱ | | | | | | | | E3 | |  |
| 37 | | ለ D3 መልስዎ አዎ ከሆነ በሳምንት ውስጥ ስንት ቀን ከ30 ደቂቃ ያላነሰ በእግርዎ ወይም በብስክሌት ይሄዳሉ? | | | | | | | | | | | | | የቀን ብዛት------------------------- | | | | | | | | E4 | |  |
| 38 | | ከፍተኛ የልብ ምት ወይም የአተነፋፈስ መጨመር ሊያስከትል የሚችል ስፖርት ቢያንስ ለ10 ደቂቃ ያክል ሳያቋርጡ ይሰራሉ? | | | | | | | | | | | | | 1. አዎ 2. አይደለም መልሱ አይደለም E8 ከሆነ ወደ ይሂዱ | | | | | | | | E5 | |  |
| 39 | | አብዛኛውን ጊዜ በሳምንት ስንት ቀን ከፍተኛ የልብ ምት ወይም የአተነፋፈስ ፍጥነት መጨመር ሊያስከትል የሚችል ስፖርት ቢያንስ ለ10 ደቂቃ ያክል ያሰራሉ? | | | | | | | | | | | | | የቀን ብዛት-------------------- | | | | | | | | E6 | |  |
| 40 | | ስፖርት ከሚሰሩባቸው ቀናት ውስጥ በአንዱ ቀን ሳያቋርጡ ለምን ያክል ጊዜ ከፍተኛ የልብ ምት ወይም የአተነፋፈስ ፍጥነት መጨመር ሊያስከትል የሚችል ስፖርት ይሰራሉ? | | | | | | | | | | | | | ሰዓት--------------------  ደቂቃ---------------------- | | | | | | | | E7 | |  |
| 41 | | መጠነኛ የሆነ ልብ ምት ወይም የአተነፋፈስ ፍጥነት መጨመር ሊያስከትል የሚችል ስፖርት ቢያንስ ለ10 ደቂቃ ያክል ሳያቋርጡ ይሰራሉ? | | | | | | | | | | | | | | | 1. አዎ 2. አይደለም | | | | | | E8 | |  |
| 42 | | አብዛኛውን ጊዜ በሳምንት ስንት ቀን መጠነኛ የሆነ የልብ ምት ወይም የአተነፋፈስ ፍጥነት መጨመር ሊያስከትል የሚችል ስፖርት ቢያንስ ለ10 ደቂቃ ያክል ሳያቋርጡ ይሰራሉ? | | | | | | | | | | | | | የቀን ብዛት------------------- | | | | | | | | E9 | |  |
| 43 | | እስፖርት ከሚሰሩባቸው ቀናት ውስጥ በአንዱ ቀን ሳያቋጡ ለምን ያክል ጊዜ መጠነኛ የሆነ የልብ ምት ወይም የአተነፋፈስ ፍጥነት መጨመር ሊያስከትል የሚችል ስፖርት ይሰራሉ | | | | | | | | | | | | | ሰዓት--------------- ደቂቃ---------- | | | | | | | | E10 | |  |
| 44 | | ከቤት ወደ ስራ ቦታ በምን ይመላለሳሉ? | | | | | | 1.በእግር2. በታክሲ/ በሰርቪስ (ባስ) 3. ሌላ ይገለፅ----------------- | | | | | | | | | | | | | | | E11 | |  |
| 45 | | አብዛኛውን ጊዜ በቀን ለምን ያክል ጊዜ (ሰዓት)ቴሌቪዥን ወይም ፊልም እያዩ ያሳልፋሉ? | | | | | | | | | | | | | ሰዓታት------------------------- | | | | | | | | E12 | |  |
| 46 | | አብዛኛውን ጊዜ በቀን እያነበቡ ለምን ያክል ቁጭ ብለው ያሳልፋሉ? | | | | | | | | | | | | | ሰዓታት------------------------- | | | | | | | | E13 | |  |
| **ክፍል አምስት፡- ስለአልኮል መጠጥ እና ሲጋራማጨስ በተመለከተ** | | | | | | | | | | | | | |  | | | | | | | | | | |  |
| 47 | | በአሁኑ ጊዜ እንደ ቢራ ያሉ አልኮል ይጠቀማሉ? | | | | | | | | | | 1.አዎ 2. አይደለም፣ መልስዎ አይደለም ከሆነ ወደ F3 ይሂዱ | | | | | | | | | | | F1 | | |
| 48 | | መልስዎ አዎ ከሆነ ምን ያህል ቀን በሳምንት ይጠጣሉ? | | | | | | | | | | | | | 1. አንድ ቀን 2. ሁለት ቀን  3. ሦስት ቀን 4. አራት ቀንና ከዛ በላይ | | | | | | | | F2 | | |
| 49 | | በአሁኑ ጊዜ ሲጋራ ያጨሳሉ? | | | | | | | | | | | | | 1. አዎ 2. አይደለም | | | | | | | | F3 | | |
| 50 | | መልስዎ አዎ ከሆነ ምን ያህል ቀን ያጨሳሉ ? | | | | | | | | | | | | | 1.በየቀኑ2. አልፎ አልፎ | | | | | | | | F4 | | |
| 51 | | በአሁኑ ጊዜ ጫት ይቅማሉ/ይጠቀማሉ? | | | | | | | | | | | | | 1. አዎ2. አይደለም | | | | | | | | F5 | | |
| 52 | | መልስዎ አዎ ከሆነ ምን ያህል ቀንይቅማሉ/ ይጠቀማሉ? | | | | | | | | | | | | | 1. አንድ ቀን3. ሦስት ቀን  2. ሁለት ቀን4.አራት ቀንና በላይ | | | | | | | | F6 | | |
| **ክፍል ስድስት:- ጤና ነክ ሁኔታዎች** | | | | | | | | | | | | | | | | | | | | |  | | | | |
| 53 | | ከአሁን በፊት ቅመም ያለው የቤተሰብ  ምጣኔ አገልግሎ ተጠቅመዉ ያዉቃሉ? | | | | | | | | | | 1. አዎ፣ አዎ ከሆነ ለምንያህል ጊዜ --------------  2.ተጠቅሜ አላዉቅም | | | | | | | | | |  | | G1 | |
| 54 | | በቤተሰብዎ ከልክ ያለፈ ክብደት ያጋጠመዉ ሰዉ አለ? | | | | | | | | | | | | | 1.አዎ 2. የለም | | | | | | |  | | G2 | |
| 55 | | መልስዎ አዎ ከሆነ የትኛዉ የቤተሰብ አባል ነውከልክ ያለፈ ክብደት ያጋጠመዉ ? | | | | | | | | | | | | | 1.አባት 2.እናት 3.ወንድም 4.እህት | | | | | | |  | | G3 | |
| 56 | | በህይወት ዘመንዎ በተደጋጋሚ ለድብርት ወይም ለጭንቀት ተጋልጠዉ ያዉቃሉ? | | | | | | | | | | | | | | | | 1.አዎ 2.የለም | | | |  | | G4 | |
| 57 | | ለረጅም ጊዜ መድሃኒት ተጠቅመዉ ያዉቃሉ? | | | | | | | | | | | | | 1. 1. አዎ 2. የለም | | | | | | |  | | G5 | |
| 58 | | መልስዎ አዎ ከሆነ መድሃኒት ለምን ህመም እንደሆነ ይገለፅ | | | | | | | | | | | | | ------------------------ | | | | | | |  | | G6 | |
| **ክፍል ሰባት:- ከመጠን ያለፈ ክብደት ግንዛቤ እና እዉቀት** | | | | | | | | | | | | | | | | | | | | | | | | | |
| 59 | | ከመጠን ያለፈ ክብደት ምክንያቶችን ያውቃሉ? | | | | | | | | | | | | | | 1. አዎ 2. አላውቅም | | | | | | | | H1 | |
| 60 | | መልስዎ አዎ ከሆነ ምክንያቶችን ጥቀሱ | | | | | | | | | | | | | | ---------------------------------  ------------------------------  ------------------------------- | | | | | | | | H2 | |
| 61 | | ከመጠን ያለፈ ክብደት ምን ጉዳት ያስከትላል? | | | | | | | | | | | | | | -------------------------------------  ----------------------------------  ---------------------------------- | | | | | | | | H3 | |
| 62 | | ክብደት/ዉፍረት መጨመር ጥሩ ነው ብለው ያስባሉ? | | | | | | | | | | | | | | ------------------------------------- | | | | | | | | H4 | |
| **ክፍል 8:- አካላዊ ልካቶች** | | | | | | | | | | | | | | | | | | | | | | | | | |
| 63 | | ወገብ | | | | በሴንቲ ሜትር | | | | | | | | | | | | | | | | | | I1 | |
| 64 | | ዳሌ | | | | በሴንቲ ሜትር | | | | | | | | | | | | | | | | | | I2 | |
